# Aerosol Concentrations and Fungal Communities Within Broiler Houses in Different Broiler Growth Stages in Summer

**DOI:** 10.3389/fvets.2021.775502

**Published:** 2021-12-13

**Authors:** Guozhong Chen, Di Ma, Qingrong Huang, Wenli Tang, Maolian Wei, Youzhi Li, Linlin Jiang, Hongwei Zhu, Xin Yu, Weibo Zheng, Jianlong Zhang, Xingxiao Zhang

**Affiliations:** ^1^School of Life Sciences, Ludong University, Yantai, China; ^2^Yantai Key Laboratory of Animal Pathogenetic Microbiology and Immunology, Yantai, China; ^3^Shandong Aquaculture Environmental Control Engineering Laboratory, Yantai, China; ^4^Shandong Provincial Key Laboratory of Quality Safety Monitoring and Risk Assessment for Animal Products, Ji'nan, China

**Keywords:** aerosols, broiler houses, fungal communities, pathogens, diversity

## Abstract

Fungal aerosols in broiler houses are important factors that can harm the health of human beings and broiler. To determine the composite characteristics and changes in fungal aerosols in broiler houses during different broiler growth stages in summer. We analyzed the species, concentration and particle diameter distribution characteristics of the aerosols in poultry houses using an Andersen sampler and internal transcribed spacer 1 (ITS1) high-throughput sequencing technology. The concentration of fungal aerosols in the poultry houses increased as the ages of the broiler increased, which was also accompanied by gradual increases in the variety and diversity indices of the fungal communities in the air of the poultry houses. During the entire broiler growth period, the dominant genera in the fungal aerosols in the poultry houses included *Trichosporon, Candida, Aspergillus, Cladosporium* and *Alternaria*. These fungi may be harmful to the health of poultry and human beings, so permanent monitoring of microbial air quality in chicken houses is necessary.

## Introduction

Microbial aerosols are suspensions of single-celled microorganisms and atmospheric particulate matter (PM) in the air, which play important roles in the transmission of pathogens (1). The microbial aerosols in poultry houses include bacteria, fungi, viruses and toxins, which are mainly from the animals themselves, their excrement, bedding and the activities of the breeders (2, 3). Along with the development of large-scale breeding industries, closed cages have been used in more broiler houses in China. However, this high density and poorly ventilated condition also leads to an increased concentration of microbial aerosols, bringing potential risks to the health of animals and human beings (4). Microbial aerosols are an important route of disease transmission, such as the avian influenza virus, and pathogen transmission may lead to outbreaks and epidemics that may cause widespread poultry morbidity or death (5). Some methods have been used to analyze biological aerosols in animal houses, such as microbial culture counting, direct microscopic detection (3, 6). In recent years, researchers have paid attention to the air pollution status of poultry rearing environments and have begun using more efficient methods, such as 16s rDNA amplicon sequencing and metagenomic sequencing, to analyze the composition of the bacterial communities; the strengths of this method is its ability to detect species that cannot be cultured, or have low abundance (7, 8). Previous reports on the fungal aerosols in poultry houses were mainly focused on colony-forming units and concentrations, while systematic studies on the fungal species present are still lacking.

Three key elements that make up the fungal aerosol hazard are the composition, concentration and particle size of the fungi in the air (9). The main mycotic diseases in broiler production include aspergillosis, candidiasis and *Fusarium*-induced osteochondrosis (10–12). Poultry houses were contaminated with high concentrations of fungal aerosols, especially in colder seasons (3). Poultry farms contain high levels of allergenic fungi, and some fungi are responsible for the development of several respiratory diseases including asthma in poultry workers (13). In a previous study, we have analyzed the changes in the composition and abundance of the bacterial community within the aerosols in closed poultry houses during the entire broiler growth cycle (8). In this study, closed-cage broiler houses will continue to be used as the research subject to collect air samples at different growth stages and analyze the type, concentration and particle distribution characteristics of fungal aerosols in the poultry houses using the Andersen sampler and internal transcribed spacer 1 (ITS1) high-throughput sequencing technology.

## Materials and Methods

### Broiler House Description

This study was carried out in three poultry houses in the Muping district of the city of Yantai in the summer of 2018. Three poultry houses located in Erjia Village (37°23′91.91^′′^N, 121°24′55.38E^′′^), Nicun Village (37°22′22.29^′′^N, 121°23′75.97E^′′^), and Dashituan Village (37°20′42.19^′′^N, 121°23′01.77E^′′^) were selected. The closed three-overlap rearing mode was used in all three houses. The roof and four sides are made of thermal insulation materials without windows. The chicken house covers an area of about 100 m × 18 m, 6 rows of chicken cages are placed, 3 layers in each row, 118 chicken cages in each layer, and 10–11 chickens in each cage. The standardized broiler houses in large-scale enterprises share the same technical standards and management system. The buildings are equipped with lamps, tunnel ventilation systems, water curtains, automatic feces transfer, a fog pipeline and coal-fired heating furnace. The air inlet of the ventilation duct is equipped with air filtering device, which can intercept 65–70% of particles above 5 μm. Three layers of chicken cages are adopted, and each layer is equipped with automatic feces conveyor. Litter and droppings were regularly cleaned every day. The reared variety was Arbor Acres (AA) broiler chickens and the density was 21.8–22.3 birds/m^2^. Each house produces 20,000–22,000 chickens during a cycle. The breeding cycle of broilers is generally 42 days. Prior to sampling, each house was cleaned and disinfected thoroughly. We chose the broiler houses where broilers were raised on the same day for the experiment, and the air samples in each house of broilers of the same growth stage were collected on the same day. Sampling times during the growth cycle were 4-day-old (with a house temperature of 30.2–31.8°C and humidity of 63%), 21-day-old (with a house temperature of 24.8–26.5°C and humidity of 59%), and 38-day-old (with a house temperature of 20.4–21.6°C and humidity of 53%) broiler chickens.

### Measurements of the Particle Diameters and Concentrations of Airborne Fungi

The air samples in each poultry house were collected using the Andersen six-stage airborne microbe sampler that met the international standard (ZR-2001, Zhongrui, Qingdao, China). The airflow rate was 28.3 L/min and the sampling time was 2 min (7, 14). Three sampling points were set equidistantly in the longitudinal direction in the middle of each poultry house at a height of 1 m. The Andersen six-stage sampler was designed and manufactured by simulating the structure of the human respiratory tract and the aerodynamic characteristics of aerosol particles. The sampler is divided into 6 stages with 400 pores in each stage, and the pore size is gradually reduced from stage 1 to stage 6. The diameters of the particles captured at each stage are >7, 4.70–7, 3.3–4.7, 2.1–3.3, 1.1–2.1, and 0.65–1.1 μm. The fungi-carrying particles in the air were captured and cultured at each stage in 9-cm glass Petri dishes with Sabouraud agar containing 0.5 g/L chloramphenicol as the sampling medium at 25°C for 72 h. Upon completion of the culture, the number of airborne fungal colonies from each sample was calculated based on the following formula, after correction according to the positive hole conversion table (1): Number of airborne fungal colonies/(CFU∙m^−3^) = Q/(0.0283 m^3^/min × T), in which Q is the total number of colonies after correction on the six Petri dishes of the Andersen six-stage sampler and T is the sampling time (1). Three samples were collected at the same time in each house, and 9 samples were collected in each growth period.

### Collection of Total Fungal Aerosols

Total fungal aerosols were collected using a Biosampler (ZR-2000, Zhongrui, Qingdao, China) in the center of the poultry house at 1.5 m above the floor. The airflow of this equipment is controllable, and the flow regulation range is 5–35 L/min. The sampling bottle (AGI-30 sampler) we selected is a special vessel for microbial sampling, working principle of which is to collect microbial particles in the air in a small amount of sampling liquid by means of jet air flow. Ultrapure water (ST876, Yaji, Shanghai, China) was used to collect samples; this water contain neither pyrogen nor nuclease (pyrogen and DNase-free water). Fifty milliliters of sampling solution (Ultrapure water) were placed in the sample bottle (airflow 12.5 L/min), and the sampling duration was 90 min (15). For gene analysis, pyrogen free can avoid the interference of microorganisms in the sampling solution, and inactivate nucleases can protect the nucleic acid in the collected microbial aerosol from the destruction of these enzymes. The collected samples were ultracentrifuged at 70,000 rpm·min^−1^ for 2 h.

### DNA Extraction and High-Throughput Sequencing

The DNA in each sample was extracted using the cetyltrimethylammonium bromide (CTAB) method and the extracted DNA was qualitatively examined by 0.7% agarose gel electrophoresis (16). The diluted genomic DNA was used as a template and amplified using the most commonly used primers for fungal ITS1 region (ITS5-1737F: 5′-GGA AGT AAA AGT CGT AAC AAG G-3′ and ITS2-2043R: 5′-GCT GCG TTC TTC ATC GAT GC-3′) (17). Polymerase chain reactions (PCRs) were performed in 30-μL volumes consisting of 15 μL of PCR Master Mix (2 ×), 0.2 μm of primers, and 10 ng of template DNA. The reaction program was 98°C for 1 min (1 cycle), 98°C for 10 s/50°C for 30 s/72°C for 30 s (30 cycles), and a final step of 72°C for 5 min. An equal amount of each PCR product was mixed evenly according to the concentration and examined by 2% agarose gel electrophoresis. The PCR products were purified using the Gel Extraction Kit (Qiagen, Germany). A DNA library was then constructed using the TruSeq^®^ DNA PCR-Free Sample Preparation Kit and sequenced on the Illumina HiSeq 2500 with 250 bp paired-end reads (PE250). All raw sequences have been deposited in GenBank under accession number PRJNA548014.

### Data Analysis

High-quality tagged data (clean tags) were obtained through strict filtering of the raw data. According to the quality control process of the QIIME software, the sequences with poor quality were removed to obtain the effective data (effective tags). The effective tags were clustered using the Uparse software and sequences with 97% consistency were automatically clustered as operational taxonomic units (OTUs), with representative sequences selected. The sequences were subjected to species annotation analysis to obtain information about each classification, such as the phylum and genus. The QIIME R software was used to calculate the Alpha and Beta diversity indices and perform differential analysis.

### Statistical Analysis

Differences in the concentrations of fungal aerosols were analyzed using GraphPad Prism 5 (GraphPad, La Jolla, USA). One-way analysis of variance (ANOVA) was employed to analyze the statistically significant differences between sample means. *P* < 0.05 was considered as statistically significant, and *P* < 0.01 or *P* < 0.001 was considered extremely statistically significant. The results are expressed as the means ± standard deviation (SD).

## Results

### Distributions of Airborne Fungi and Diameters of Aerosol Particles

**Figure 1:**
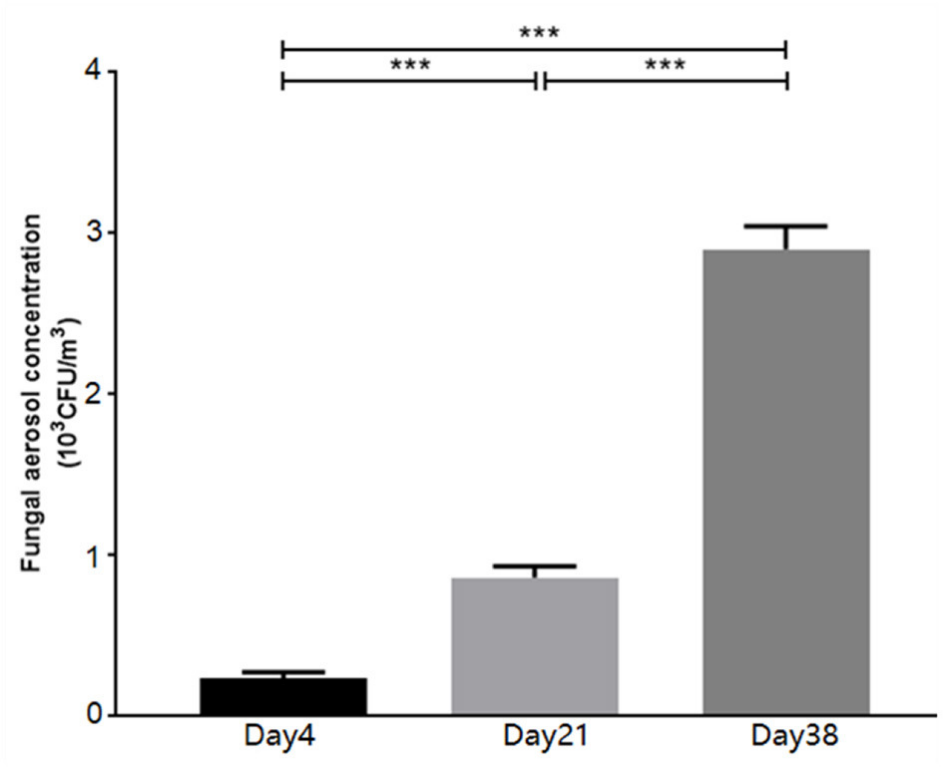
The concentration of airborne fungi in the poultry houses measured at different stages using the Andersen six-stage sampler. Data are expressed as the means ± SDs, ^***^P < 0.001 compared between the two groups (*n* = 9).

The concentrations of fungal aerosols in the poultry houses at different broiler growth stages are shown in Figure 1. At 4 days old (Day 4), the fungal aerosol concentration in the poultry houses was 0.23 ± 0.02 × 10^3^ CFU/m^3^, while the concentration was 0.86 ± 0.04 × 10^3^ CFU/m^3^ at 21 days old (Day 21) and 2.90 ± 0.08 × 10^3^ CFU/m^3^ at 38 days old (Day 38). Compared to Day 4, the fungal aerosol concentration in the poultry houses on Day 21 was significantly elevated (*P* < 0.001). Compared to Day 21, the fungal aerosol concentration in the poultry houses on Day 38 was significantly elevated (*P* < 0.001).

**Figure 2:**
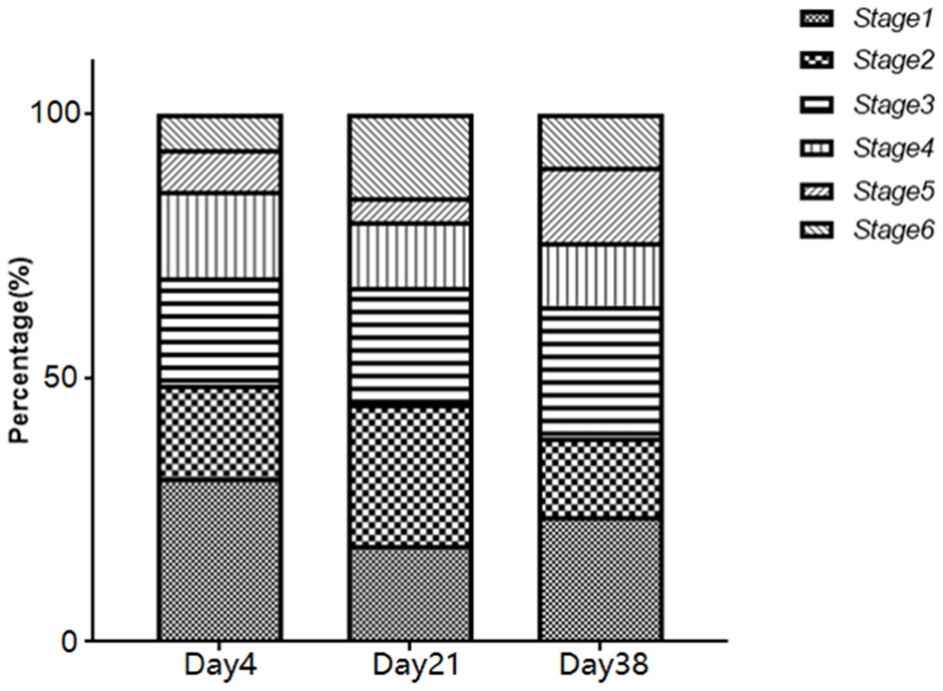
Distribution ratio of airborne fungi on different stages of the Andersen six-stage sampler.

The distribution of the fungal aerosol particle diameters in the poultry houses during different growth stages are shown in Figure 2. In the samples from Day 4, the fungi were mainly distributed to stage 1 (31.2%), with the lowest distribution on stage 6 (6.5%). In the samples from Day 21, the fungi were mainly distributed to stage 2 (25%), with the lowest distribution on stage 5 (4.3%). In the samples from Day 38, the fungi were mainly distributed to stage 1 (23.8%) and stage 3 (25.1%). Additionally, in the samples from Day 21 and Day 38, the number of fungi distributed to stage 5 and stage 6 (0.65–2.1 μm) was significantly higher than that of Day 4 (*P* < 0.05).

### Sequencing Statistical Analysis

Sequencing of the genes in the fungal ITS1 region from the air of the poultry houses revealed a total of 706,444 raw sequences in the sample (raw PE). After filtering out the sequences with poor quality, 657,946 effective tags were obtained (among which, 186,326 were from the air in the poultry house on Day 4, 199,047 were from Day 21, and 272,573 were from Day 38). The effective tags from the 9 samples clustered together with 97% identity and 2,946 OTUs were obtained that annotated 178 genera. Additionally, the dilution curve for all samples was almost saturated, suggesting that the majority of fungi in the samples were detected, ensuring the reliability of the subsequent analysis.

### Diversity of Fungal Communities

**Figure 3:**
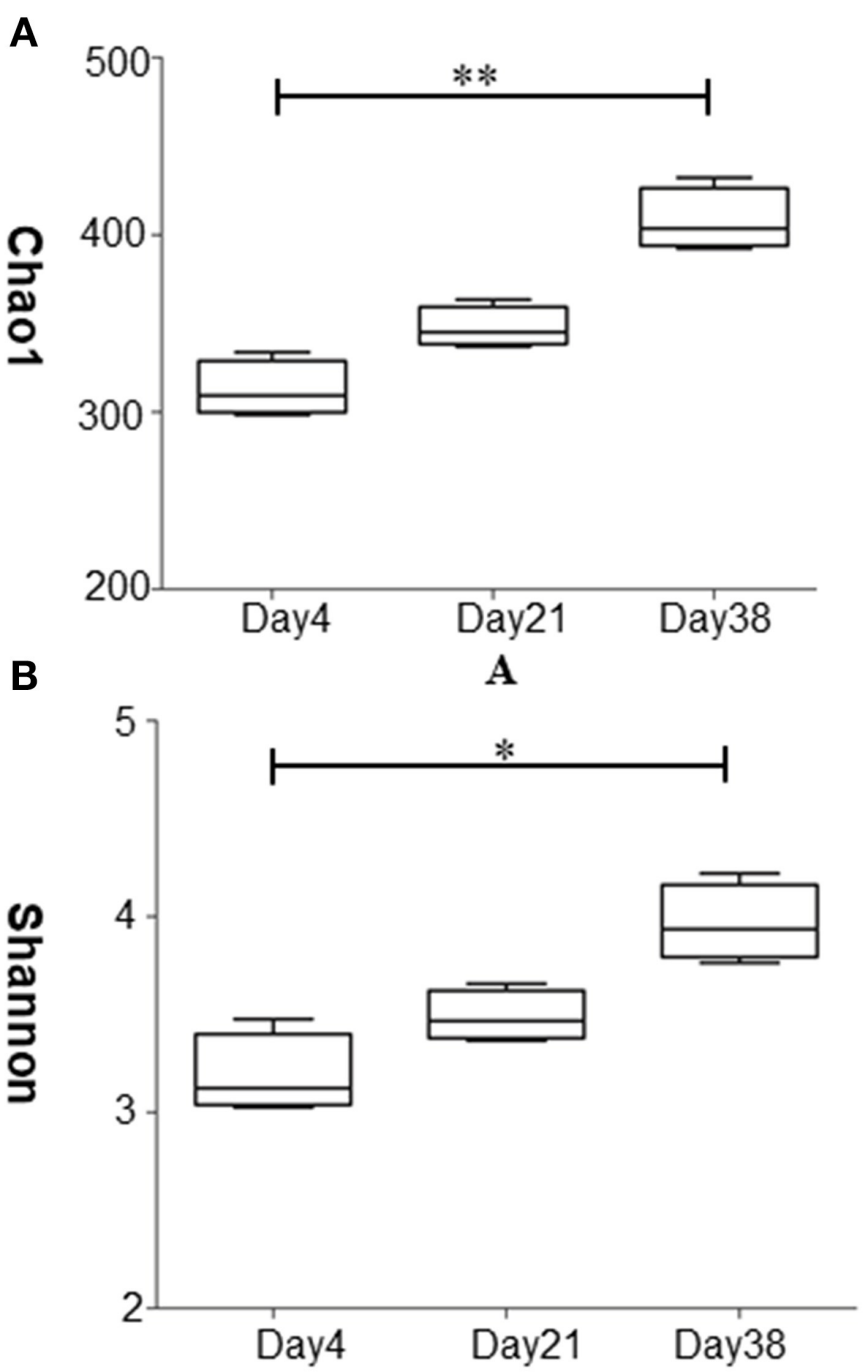
Analysis of the diversity of fungal community at different stages in the poultry house. **(A)** Chao1 diversity of the fungal community. **(B)** Shannon diversity of the fungal community. Data are expressed as the means ± SDs, ^**^P < 0.01, ^*^P < 0.05 compared between the two groups (*n* = 3).

The abundance and diversity of microbial species in the samples were assessed by α diversity indices including the Chao1 index and the Shannon index (Figure 3). The results of the Chao1 index are shown in Figure 3A. The Chao1 indices of the samples from Day 21 and Day 38 were both higher than that of Day 4, with the Chao1 index of Day 38 significantly higher than that of Day 4 (*P* < 0.01), indicating that the fungal abundance was highest in the air at the late stage of broiler growth. Figure 3B shows that the Shannon index was highest on Day 38 and was significantly higher than those of Day 4 (*P* < 0.05), indicating that the fungal community in the air was most diverse during the late stage of broiler chicken growth.

**Figure 4:**
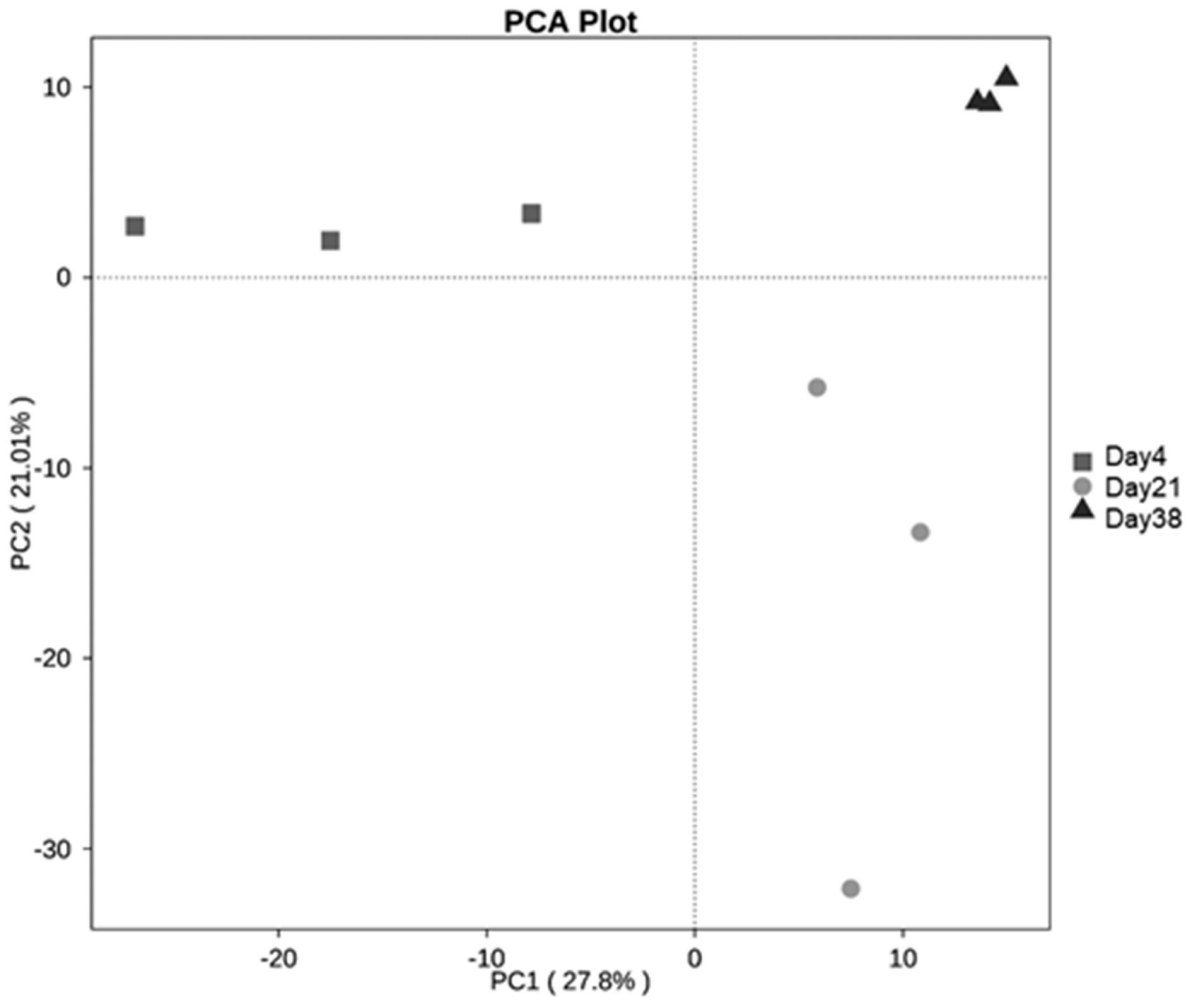
PCA analysis based on the OTU levels of each sample. PC1 represents one principal component, PC2 represents another principal component, and the percentages represent the contribution of the principal components to the sample differences.

In the β diversity analysis, two coordinate axes that best reflected the differences among the samples were extracted using the principal component analysis (PCA), the more similar the community composite of the samples, the closer they are on the PCA graph. The PCA analysis results based on the OTU levels (Figure 4) showed that there were some differences among the fungal communities of the three groups of samples. The PCA analysis in combination with the multiple response permutation procedure (MRPP) test showed that Day 4 and Day 38 differed significantly (*P* < 0.05).

### Fungal Community Composition

**Figure 5:**
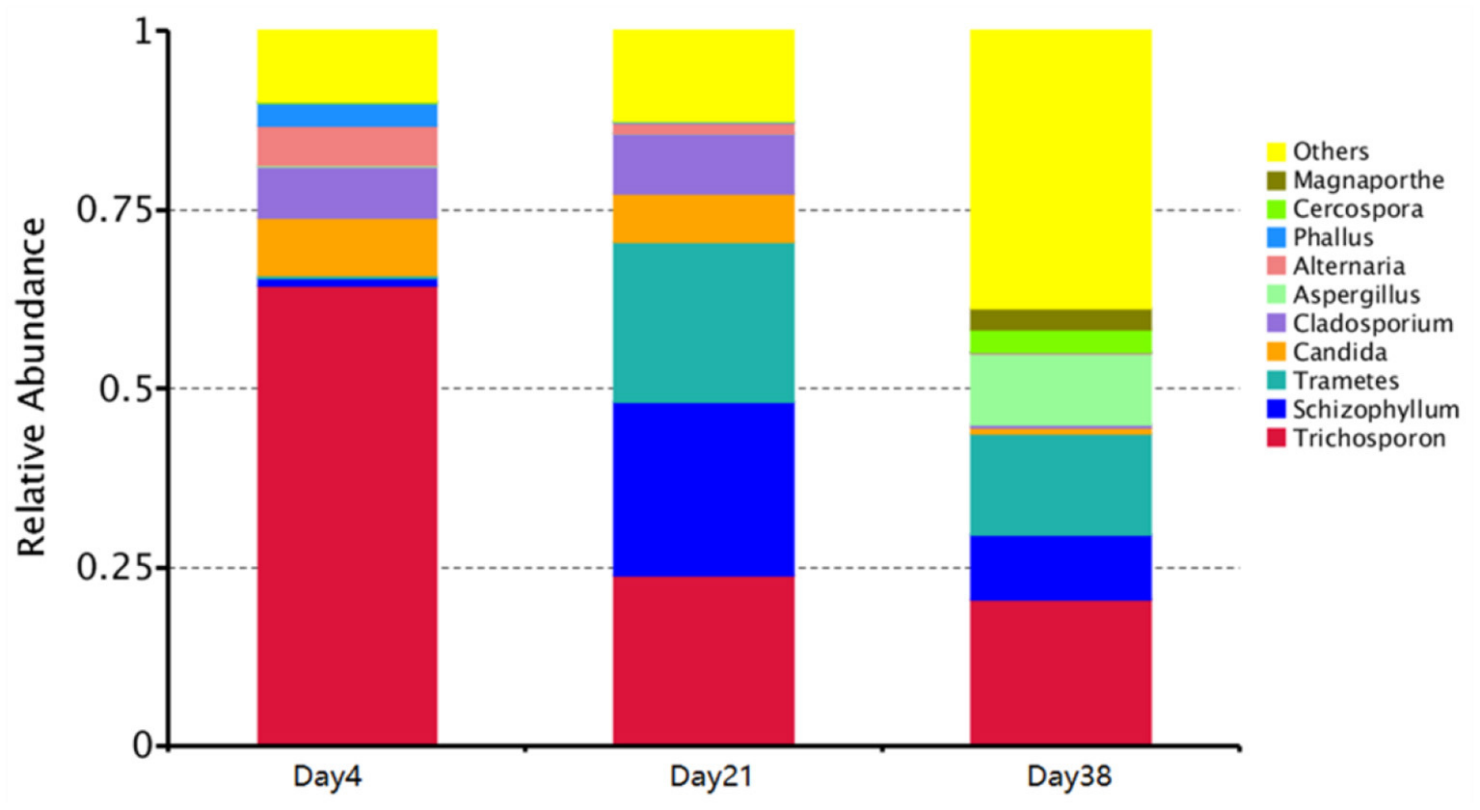
Graph of the relative species abundance of each sample at the genus level. X-axis indicates each sample and Y-axis indicates relative abundance.

The phyla with the highest contents in all fungal aerosol samples were the Basidiomycota and Ascomycota, accounting for 73.27 and 25.79% of Day 4 samples, 78.46 and 21.14% of Day 21 samples, and 50.36 and 49.63% of Day38 samples, respectively. Figure 5 shows the top ten genera with the highest abundance in the samples. Our data showed that the dominant fungal genera in the Day 4 samples were *Trichosporon* (64.35%), *Candida* (7.96%), *Cladosporium* (7.13%), *Alternaria* (5.63%), *Trametes* (3.84%) and *Phallus* (3.00%). The most abundant fungal genera in the Day 21 samples were *Schizophyllum* (24.33%), *Trichosporon* (23.90%), *Trametes* (22.22%), *Cladosporium* (8.33%), *Candida* (6.86%) and *Alternaria* (1.63%). The most abundant fungal genera in the Day 38 samples were *Trichosporon* (20.40%), *Trametes* (14.01%), *Aspergillus* (9.89%), *Schizophyllum* (9.27%), and *Cercospora* (3.27%).

**Figure 6:**
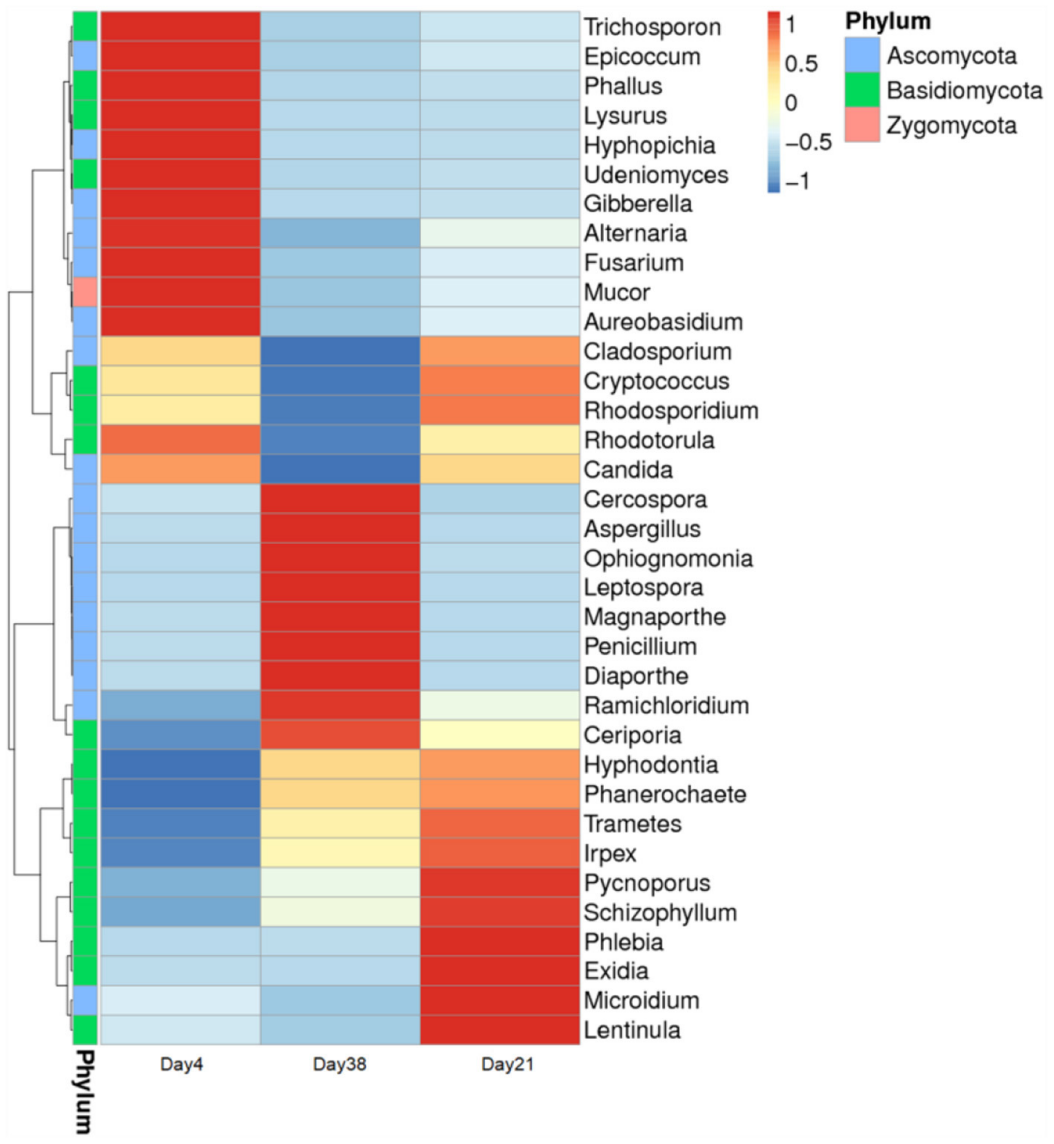
Clustering map of the fungal community species abundances at the genus level in the poultry houses at different broiler chicken growth stages. The sample information is shown crosswise, and the species annotation is shown lengthwise. The species cluster tree is shown on the left.

A heat map of the phylogenetic tree was constructed based on the 35 different genera that could be classified (Figure 6). Each grid in the figure represents the relative abundance of one fungal species at the genus level with darker colors indicating higher abundances. Our results showed that at different broiler chicken growth stages, although the fungal community compositions in the air inside the poultry house were largely the same, their abundances changed to a certain extent.

## Discussion

The broiler houses in this study are in the suburbs, the air is fresh, and the filter ventilation device is adopted. Therefore, it can be predicted that the fungal concentrations in the air entering the chicken house are very low. Frequent ventilation significantly reduces the fungal concentration in indoor air, which has been confirmed by past studies. Many studies have reported on the air pollution inside and outside the chicken house caused by fungi and particles. Researchers found that the concentrations of indoor fungi and particles were higher than those of outdoor air, and the species of indoor and outdoor fungi were different (18). Although some fungi appear in indoor and outdoor air at the same time, there is no evidence that indoor fungal pollution comes from outdoor. On the contrary, many researchers believe that the farming buildings are emitters of the considerable amounts of microbiological contaminants into the atmospheric air, and this high emission of potentially pathogenic microorganisms via aerosols from animal housing facilities to the outdoor environment may constitute a considerable risk to human health and environmental pollution (19). The propagation distance of airborne microorganism in farming environment is generally considered to be <1,000 m under the conditions of downwind or upwind (20, 21). The distance between each broiler house is more than 3,000 m, so there was no interaction between the measured bioaerosol concentrations or species spectra in the houses.

Our study showed that the concentration of fungal aerosols increased as the broilers aged, which is consistent with the changes in the atmospheric particulate matter (PM2.5 and PM10) (8). The airborne fungal concentration was lowest on Day 4 and increased significantly on Day 21 and Day 38 (*P* < 0.001). This is because along with the growth of the broiler chickens, their increased activities and excretion, as well as a relatively higher housing density, lead to increased concentrations of fungal aerosols in the poultry houses. A previous study showed that window ventilation could reduce fungal aerosol concentrations in poultry houses, with increased ventilation reducing of the incidence of mycosis by 75% (22). This indicated that fungal contamination of the rearing environment can be improved by human factors.

We also assessed the distribution of fungal aerosol particles of different sizes in the poultry houses using the Andersen six-stage sampler, which is suitable for the respiratory tract research of most animals. It can also be used in the respiratory tract research of broilers. The respiratory system of birds includes nostrils, nasal cavities, larynx, trachea, cavities, main bronchus (middle bronchus), second bronchus, counter bronchus (accessory bronchus), capillaries, lungs and air sacs, the basic structure of which is similar to that of human respiratory system. The aerosol particles collected on stages 1–4 of the Andersen six-stage sampler can enter the respiratory tract of human beings and animals through the nasal cavity and pharynx, however, a portion of the particles can be cleared from the body through mucociliary escalation, causing minimal harm to health (23). The particles collected on stages 5–6 can directly enter the deepest sections of the respiratory tract, the bronchi and pulmonary alveoli, and they are not easily cleared, leading to serious impacts on the health of human beings and animals (24). Such as Inhalation of *Aspergillus* (*Aspergillus fumigatus*) spores (ϕ ≈ 2–3 μm) by broilers can directly enter the deepest sections of the respiratory tract, which can produce severe inflammation in lungs, air sacs and sometimes other tissues (25). The results showed that the distribution of fungal aerosols in the air of the poultry house to stages 5–6 during the middle (Day 21) and late (Day 38) growth phases was significantly higher than that of the early phase (Day 4), indicating that airborne fungi in the poultry house may pose a more serious threat to the health of the chickens in the middle and late growth phases. Therefore, smaller aerosol particles should be used during the disinfection of poultry houses with atomization devices.

Using ITS1 amplicon sequencing technology, our study identified fungi from 5 phyla and 178 genera, which was far fewer than the number of bacteria identified previously. Additionally, the α diversity indices of the airborne fungal communities were less than those of the bacterial communities during the same period of time (8). Chien et al., also found that the fungal concentrations and species detected in the bioaerosols of animal houses were far less than those identified for bacteria (4). We also found that with the increasing age of birds, the α diversity indices of the fungal community in the poultry house air gradually increased. The Chao1 index and the Shannon index on Day 38 were significantly higher than on Day 4 (*P* < 0.05). PCA analysis also showed significant change in the composition of the fungal community (*P* < 0.05). With the growth of the chickens, increased activities, changes in metabolism levels, increased excretion, and relatively higher housing densities all lead to changes in the microbial community structure in the air of the poultry houses (26).

At the phylum level, the Basidiomycota and Ascomycota accounted for the majority of fungi in the air of poultry houses. The Basidiomycota are widely distributed, present in almost all terrestrial ecosystems, and large in number and variety, including many beneficial fungi that can be used for medication and food, as well as many that are pathogenic to plants and animals (27). The Ascomycota is the largest fungal group with members that are mostly terrestrial. Some parasitic ascomycetes, such as some *Penicillium* and *Aspergillus* species, cause not only plant diseases but also diseases in humans, livestock, and poultry (28). It was found that the most fungi in chicken houses in summer were *Candida* (accounting for more than 69%), followed by *Penicillium, Aspergillus, Cladosporium, Alternaria*, and *Fusarium* (19). Similarly, these fungi were relatively abundant in this study (Figures 5, 6). *Schizophyllum, Trametes, Phallus* and other fungi found in this study have not been found and isolated in poultry houses before. Gene detection technology can not only find fungi with high content in the air of poultry houses, but also find some fungi with low content. However, some fungi may only be dead spores, dead cells or DNA fragments and are not infectious. Next, we will conduct culture research on the collected air particulate matter samples to distinguish between dead and living cells.

The respiratory system of chickens is an “open” structure from nose to lung, abdominal organs, bones and related tissues. Therefore, respiratory tract infection tends to occur and spread to abdominal organs and even the whole body. Although the period of chicken rearing is very short, broilers are very likely to be infected with fungi because of the high concentration of fungal aerosol in the poultry house. The dominant fungi with high abundances included *Trichosporon, Candida, Aspergillus, Cladosporium* and *Alternaria*. Among these, *Trichosporon* was highly abundant in all stages. A study showed that the dietary addition of different concentrations of *Trichosporon mycotoxinivorans* (i.e., 10^4^ CFU/g of feed, 10^5^ CFU/g, 10^6^ CFU/g) completely blocked the adverse effects of ochratoxin A on several immune characteristics of broiler chicken (29). However, the *Trichosporon* genus also contains some pathogenic strains that are harmful to the health of humans and animals, such as *Trichosporon asahii* (30). Although some researchers believed that the soil around the farm or poultry manure was the main source of *Trichosporon* (31), in this study, most of them may come from the feed and feces of broilers because the chicken houses adopted air filtration system. We also detected the presence of *Candida* (Day 4: 7.60%, Day 21: 6.86%, Day 38: 0.81%). *Candida* is the most common pathogenic fungi causing diseases in poultry, such as infection with *Candida albicans*, which a common mycosis of the digestive tract that is manifested by dyspepsia, reduced feeding, reduced feed conversion ratio and even death (32). Avian aspergillosis is one of the mycoses that are most harmful to the poultry industry and can be detected in many poultry houses. This disease is caused by *Aspergillus* species and *Aspergillus fumigatus* is believed to be the most pathogenic species (33). We detected the presence of *Aspergillus* in all air samples and on Day 38 this fungal genus accounted for 9.89%, which may cause serious damage to the health of broiler chickens. Additionally, we also detected *Cladosporium* and *Alternaria* in the poultry houses at different stages in this study, with these genera being highly abundant in the early and middle growth phases of the broiler chicken. Some previous studies also showed the widespread presence of these fungi in the air of poultry farms (34, 35). Microbial pollutants in the chicken house may be discharged to the outdoor environment through aerosols. Additionally, some fungal strains of *Cladosporium* and *Alternaria* can cause respiratory diseases in humans and animals (35, 36).

Some researchers believe that initial contamination of poultry farms may occur through use of a moldy litter or introduction of one-day-old birds whose down has retained conidia in hatchery facilities (37). Pollution sources of airborne fungi may include feed, dust, feces, skin and outer air (38, 39). Acute aspergillosis generally occurs in young birds resulting in high morbidity and mortality. The chronic form causes lesser mortality and generally affects older birds, especially breeders in poultry, presenting a compromised immune system due to poor husbandry conditions (37, 40). In addition, we found that the higher the concentration of particulate matter in poultry houses, the higher the fungal content, and the greater the possibility of broiler disease. Further, we will study the effects of fungal aerosols in poultry houses on the health of broilers and the pathogenesis.

This study indicates that with increasing broiler chicken age, the concentration of fungal aerosols, the species and diversity index of the fungal community in the air of the poultry houses increases gradually. During the entire growth process, the dominant genera in the fungal aerosols of the poultry houses include *Trichosporon, Candida, Aspergillus, Cladosporium* and *Alternaria*; these fungi may be harmful to the health of poultry and even humans and should be paid attention to. As the age of the chickens increase, personnel should clear the garbage inside poultry houses in a timely manner, keep the humidity appropriate, strengthen the ventilation, and disinfect with disinfectants that are effective against both bacteria and fungi, which will provide a friendlier environment for poultry growth. For the health of people and poultry, air samples should be collected every few days during the rearing process to detect air particulate matter and fungal communities. However, at present, the detection methods of pathogenic microorganisms in poultry breeding take too long and have poor timeliness. Rapid detection kits should be developed for common pathogens causing poultry diseases. Only in this way an antidote against the disease can be found. This will play an important role in epidemiological research. Our study emphasizes the necessity for permanent monitoring of microbial air quality of chicken houses.

## Data Availability Statement

The datasets presented in this study can be found in online repositories. The names of the repository/repositories and accession number(s) can be found below: https://www.ncbi.nlm.nih.gov/genbank/, PRJNA548014.

## Ethics Statement

The animal study was reviewed and approved by Animal Care and Use Committee (ACUC) of the School of Life Sciences, Ludong University (SKY-ACUC-2018-07).

## Author Contributions

JZ and XZ designed the research. GC and DM performed the experiments. LJ, XY, HZ, and QH analyzed data and contributed figures and tables. JZ and MW wrote the article. WT, YL, and WZ advised on the manuscript content and reviewed the manuscript. All authors read and approved the final manuscript.

## Funding

This research was funded by the National Key Research and Development Program of China (Grant No. 2018YFD0501402), the Major Agricultural Applied Technological Innovation Projects of Shandong Province (to XZ), the Key Research and Development Plan of Yantai (No. 2018XSCC045), and the Innovation Team Project for Modern Agricultural Industrious Technology System of Shandong Province (SDAIT-11-10).

## Conflict of Interest

The authors declare that the research was conducted in the absence of any commercial or financial relationships that could be construed as a potential conflict of interest.

## Publisher's Note

All claims expressed in this article are solely those of the authors and do not necessarily represent those of their affiliated organizations, or those of the publisher, the editors and the reviewers. Any product that may be evaluated in this article, or claim that may be made by its manufacturer, is not guaranteed or endorsed by the publisher.
